# Write-up and dissemination of undergraduate and postgraduate research at the University of Rwanda: a cross-sectional study

**DOI:** 10.11604/pamj.2019.32.164.18409

**Published:** 2019-04-09

**Authors:** Christian Nsanzabaganwa, Hubert Habineza, Naphtal Nyirimanzi, Christian Umuhoza, Katie Cartledge, Craig Conard, Peter Cartledge

**Affiliations:** 1 College of Medicine and Health Sciences (CMHS), University of Rwanda, Kigali, Rwanda; 2 Department of Pediatrics, Univerisity Teaching Hospital of Butare, Butare, Rwanda; 3 Department of Pediatrics, University Teaching Hospital of Kigali, Kigali, Rwanda; 4 School of Medicine, Yale University (New Haven, USA), Rwanda Human Resources for Health (HRH) Program, Kigali, Rwanda

**Keywords:** Research, internship and residency, writing style, education, medical, developing country

## Abstract

**Introduction:**

Research is essential in all areas of health development. However, medical students and residents frequently lack the time and training on performing research. This is especially prevalent in resource-limited settings. We aimed to compare the word counts of undergraduate and postgraduate dissertations with published projects in Rwanda, and to identify the proportion of postgraduate pediatric research projects that have been published since 2012.

**Methods:**

Retrospective, cross-sectional study of undergraduate and postgraduate research dissertations at the University of Rwanda. Dissertations were then compared to randomly selected published papers of Rwandan research. Each IMRaD (Introduction, Methodology, Results and Discussion) section word count was compared using Student's t-test.

**Results:**

19/190 (10%) undergraduate dissertations and 22/41 (54%) postgraduate dissertations, were available in electronic format for word-count analysis. The mean total word count for postgraduate dissertations (5163 words) was significantly longer (p<0.001) than the randomly selected peer-reviewed journal articles (2959 words). Each section of the IMRaD structure of postgraduate dissertations was significantly longer than those of the control group. Undergraduates used a similar number of words to published papers, but used significantly more tables and figures. Of the 41 postgraduate dissertations, only four (10%), were published in peer-reviewed journals.

**Conclusion:**

This is the first study to assess the writing style of Rwandan medical students and pediatric postgraduate residents. A simple step to increase dissemination of research findings would be for institutions to modify academic regulations so that students write-up in manuscript form rather than dissertation format.

## Introduction

Research is vital in different avenues of health development and care delivery whilst also playing an essential role in the academic advancement of the researcher [1-3]. Research is defined as “critical and exhaustive investigation or experimentation, having for its aim the discovery of new facts and their correct interpretation, the revision of accepted conclusions, theories, or laws in the light of newly discovered facts, or the practical application of such new or revised conclusions, theories, or laws” [4]. It should, therefore, be part of the educational culture in medical training programs [5]. There are benefits to both medical students and residents, who shall be referred to as 'students' in this article. It is now evident that engaging students in research may lead to increased participation in research after completion of training [6, 7]. However, due to the burdens of patient care, students are frequently too busy to find sufficient time for research activities [8, 9]. Scholarly research activity programs are essential components of the modern undergraduate (UG) and postgraduate (PG) medical curriculum [10]. Research encourages students to develop the necessary competencies for future research activities and enter academic careers as physician-scientists [11]. Undertaking research can help develop transferable skills such as communication skills, time management, medical statistics, academic writing, systematic thinking, critical appraisal skills, information technology (IT) skills and how to practice evidence-based medicine [3, 10]. In Rwanda, medical students and residents face various barriers to performing research, notably a lack of good mentorship and financial burdens; yet they have to undertake research in order to graduate from their training [5]. It is an aim of the University of Rwanda (UR) to improve the quality and quantity of their research output [12]. In 2014, thanks to intervention from the broader community, Rwanda was the most influential country for research in East Africa for published research output, with the UR being the second most influential University in East Africa [12].

Peer-reviewed published papers (control group) classically follow the IMRaD structure of Introduction, Methodology, Results and Discussion [13]. Undertaking a research dissertation is already a significant challenge to students. Therefore, progressing to the submission of the results in a peer-reviewed journal adds a new burden to the student's research journey and requires its own specific competencies. The language used in “writing-up for publication” can be foreign to the student and many students in this setting have had little opportunity or feedback in academic writing. One study in Rwanda demonstrated that students needed formal training to develop these skills in order to improve the scholarly output of the participants [14]. To compound this, many academic institutions ask students to submit research in the form of a “dissertation” which may have other idiosyncrasies which are not consistent with manuscripts in peer-reviewed journals and dissertations may have specific requirements related to the institution. Publication is defined as “copies of a work or document distributed to the public by sale, rental, lease, or lending” [4]. In this study, we define publication as being articles that have gone through peer-review by a medical journal. Encouraging students to publish dissertation work is important as the future synthesis of research, in systematic reviews, is compromised if journal publications represent a biased selection of research projects conducted [15]. Many research projects, however, go unreported with one study finding that only 46% of US NIH-funded trials were published in a peer-reviewed, Medline-indexed journal within 30-months of completion [16]. Furthermore, the research work represents not only the author but also the research institution, the funding body, and other researchers [17]. Submission for publication can equip the student in quality scientific writing, presentation skills and raising their knowledge and practice of scientific ethics [18]. Writing-up research in a concise manner is a fundamental step in the dissemination process. There is currently no data on the the conciseness of writing style of UG and PG research projects undertaken in Rwanda or on the number of these projects published in peer-review journals. Aims: this research project aimed to identify correlation between peer-reviewed published papers (control group) and undergraduate (UG) or postgraduate (PG) projects in Rwanda, and to identify what proportion of resident research projects have been published since 2012. We hypothesized that the word count of the IMRaD sections of UG and PG dissertations would be significantly longer than randomly identified peer-reviewed published research manuscripts from Rwanda.

## Methods

**Study design:** A retrospective cross-sectional study of undergraduate (UG) and postgraduate (PG) dissertations (monographs) compared to similar peer-reviewed published papers (control group). Reporting of the current study is in accordance with the STROBE (Strengthening the Reporting of Observational Studies in Epidemiology) checklist [19].

**Study setting and location of the population:** The University of Rwanda (UR) is a singular, multi-campus institution established in 2013 from the merger of the nation's seven public Higher Learning Institutions (HLIs) into a single, public university with six self-governing colleges, of which the College of Medicine and Health Sciences (CMHS) is a part [12]. All final year medical students and MMed (master of medicine) pediatric residents at the UR are required to undertake a research project and write this as a research dissertation (monograph) as a condition of graduation [20].

### Participants/subjects

**Inclusion criteria:** Group 1: research projects of final year undergraduate (UG) medical students (interns) who graduated between 2016-2017 (n=190); group 2: research projects of pediatric postgraduate (PG) MMed residents who graduated between 2012-2017 (n=41); group 3 (control group): peer-reviewed published papers (n=22). Random research publications with the keyword “Rwanda” were opportunistically identified by searching the websites of the following peer-reviewed journals: Malawi Medical Journal (MMJ); East African medical journal (EAMJ); Pan-African medical Journal (PAMJ); Tropical Medicine International Health (TMIH); Journal of Tropical Pediatrics (JTP); Rwandan Medical Journal (RMJ); Paediatrics and International Child Health (PICH). These journals were chosen because of their relevance to our setting.

**Exclusion criteria:** Dissertations only available in paper (hard) format.

**Recruitment/enrolment of participants:** *Recruitment (Group 1 and Group 2):* dissertations performed at UR remain the copyright of UR [**20**] and therefore should be available. Therefore, dissertations were obtained from several sources: i. An email was sent to all previous students in groups 1 and 2 inviting them to send their completed dissertations; ii. We contacted UR faculty who had supervised students undertaking research asking them to send the dissertations of their students; iii. The UR main library at the School of Medicine was manually searched; iv. The pediatric department holds a database of all the pediatric PG graduates and research topics. Finally, the dissertations fulfilling criteria were selected and enrolled in the study.

**Variables and outcomes:** The primary outcomes were word counts of each of the IMRaD sections used in writing up dissertations. Secondary outcomes were details of the projects performed, namely; study methodology, site, retrospective/prospective and finally, using our database of pediatric PG graduates and topics (2012-2017, n=41) to identify whether the research projects were published in a peer-review journal. To identify whether articles had been published, we searched PubMed, Scopus and Google, alongside networking with local graduates and academic staff. We did not attempt to identify the proportion of UG projects published. Current academic regulation at the University of Rwanda requires that memories be limited to 300 words for the abstract and 15,000 words for the entire body of work (including appendixes, cover pages, references etc). The author instructions of the seven peer-reviewed journals were reviewed and the average maximum word count was 3,700 (3500-4000), excluding tables and references etc.

**Power calculation:** We recruited using convenience methods. However, a post-hoc power calculation was performed using G*power software. The primary outcome was a comparison of the total word count of the UG and PG dissertations with the control group (peer-reviewed published papers). Based on our results, the power calculation for independent t-tests (compared to peer-reviewed published papers) was 0.99 for PG dissertations and 0.30 for the UG dissertations compared to the control. Therefore, the UG arm of the study was underpowered to find if there were any significant difference between the groups.

**Data collection and management:** For all dissertations that were available in electronic form, we calculated the word counts of abstracts, introduction, methods, analysis, results, and discussion using the “word count” function in Microsoft Word, as well as manually counting the number of tables and figures of each paper. Statistical Package for the Social Sciences (SPSS) was employed for statistical analysis.

**Statistical analysis:** *Categorical variables* were described using descriptive statistics and analyzed using Chi-squared. *Continuous (scale) variables* were described using means and standard deviations and analysed using Student's t-tests for independent variables.

**Ethical considerations:** No social, emotional, physical, legal, or financial ethical risks to participants were identified. Publishing in a peer-review journal is not a requirement of graduation therefore there is no implication on the authors. Memoirs remain the copyright of UR and therefore should be available for the furthering of scientific knowledge of the academic community. As dissertations remain the copyright of the UR, no consent was gained. To ensure confidentiality, all information gathered was kept securely in a password-protected spreadsheet. Only the investigator of this project and the supervisors had access to the information kept in the database. Research protocol was reviewed and approved by UR CMHS Institution Review Board (IRB) on the 27 July 2017 (Ref: 341/CMHS IRB/2017 and 340/CMHS IRB/2017).

**Availability of data and materials:** Non-analyzed datasets used and/or analyzed during the current study are available from the corresponding author on reasonable request.

## Results

**Figure 1:**
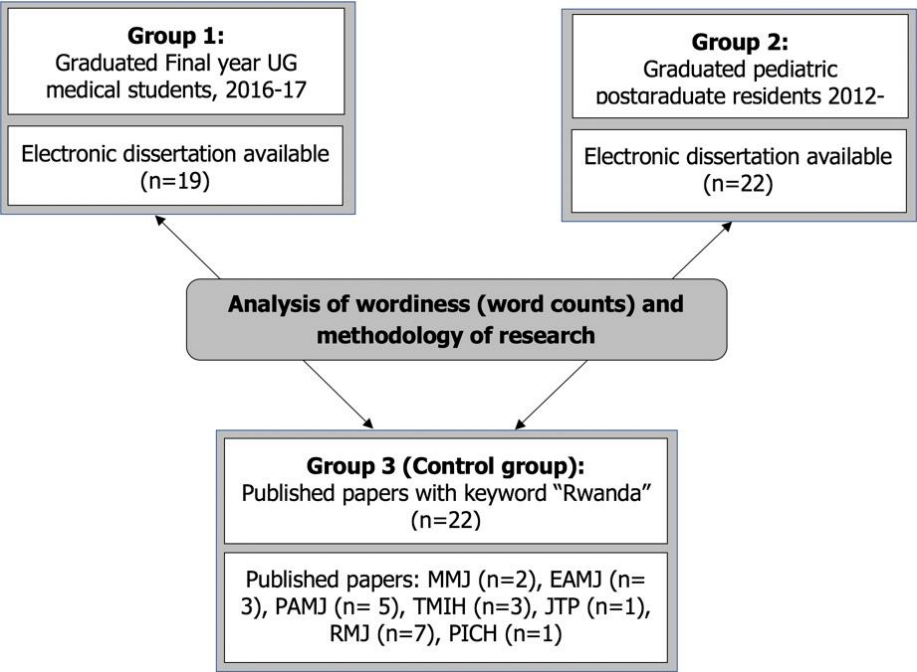
Group recruitment

**Baseline data on dissertations performed:** We were able to access 19/190 (10%) UG and 22/41 (54%) PG resident dissertations in electronic format for analysis of word counts (Figure 1). Five were available in hard-print format but were excluded as they were not suitable for word-count analysis on Microsoft Word. Twenty-two peer-reviewed published articles were found from MMJ (n=2), EAMJ (n= 3), PAMJ (n= 5), TMIH (n=3), JTP (n=1), RMJ (n=7), PICH (n=1). “Non-participants” were those dissertations that were not available despite the steps taken in the methodology.

**Methodology of research employed:** All the research undertaken by the UG and PG students was clinical research. Cross-sectional research was performed frequently in all three groups. UG students were much more likely to undertake retrospective research than their PG counterparts (Table 1).

**Table 1:** Baseline data on dissertations and peer-reviewed articles

	Group 1: Undergraduate Dissertations (n=19)	Group 2: Postgraduate Dissertations (n=22)	Group 3: Peer-reviewedPublished papers (n=22)	Total (n=63)
**Timing of project**				
Retrospective	17(89.5%)	0(0.0%)	7(31.8%)	24(38.1%)
Prospective	1(5.3%)	21(95.5%)	15(68.2%)	37(58.7%)
Mixed	0(0.0%)	1(4.5%)	0(0.0%)	1(1.6%)
Unknown	1(5.3%)	0(0.0%)	0(0.0%)	1(1.6%)
**Methodology employed**				
Cross-sectional study	17(89.5%)	16(72.7%)	13(59.0%)	46(73.0%)
Cohort study	0(0.0%)	0(0.0%)	3(13.6%)	3(4.8%)
Case-control study	2(10.5%)	1(4.5%)	0(0.0%)	3(4.8%)
Other	0(0.0%)	5(22.7%)	6(27.3%)	11(17.5%)

**Publishing of PG research projects:** Of the research dissertations of pediatric PG residents who graduated between 2012-2017 (n=41), we found evidence of four dissertations (10%) that had been published in peer-review journals.

**The writing style (distribution of word counts in UG and PG research):** Data was extracted from digitally available dissertations (Table 2) and peer-reviewed published papers. No data-points were missing. The mean total word count (excluding abstract, tables, figures and references) for peer-reviewed published articles was 2959 words with a mean of 4 tables/figures. The mean total word count for PG dissertations was 5163 which was significantly larger (p<0.001) than the peer-reviewed published papers. Each section of the IMRaD structure, of PG dissertations, was significantly longer than those of peer-reviewed articles. Interestingly PG dissertations (77%) were more likely to present keywords than control group (46%). UG dissertations did not significantly differ from the control group (published papers) except for the number of tables and figures which were significantly larger (p<0.001). We observed that this large number of tables reflected students' tendency to copy and paste tables from SPSS rather than to consolidate data into summary, tables.

**Table 2:** Distribution of text, figures and references for intern, resident and published papers

	Group 3 (control group): Peer-reviewed Published papers (n=22)	Group 1: Undergraduate Dissertations (n=19)	p-value^t^ (df=42)	Group 2: Postgraduate Dissertations (n=22)	p-value^t^ (df=46)
Mean **total word count** (excluding abstract, tables, figures and references)	2959 (±1224)	3518 (±1274)	p=0.148	5163 (±2048)	p<0.001
Mean **abstract** word count	225 (±71)	260 (±160)	p=0.351	357 (±128)	p=0.001
Mean **introduction**word count	445 (±214)	1382 (±639)	p=0.351	1170 (±534)	p<0.001
Mean **methodology** word count	631 (±378)	502 (±211)	p=0.192	1123 (±487)	p<0.001
Mean **results** word count	888 (±758)	1045 (±488)	p=0.448	1372 (±873)	p=0.048
Mean **discussion** word count	995 (±463)	680 (±579)	p=0.054	1732 (±684)	p<0.001
Mean **number of tables and figures**	4 (±3)	13 (±6)	p<0.001	7 (±3)	p=0.011
Mean **number of references**	26 (±14)	20 (±9)	p=0.163	37 (±15)	p<0.001
Were **Keywords**presented	10/22 (45.5%)	5/19 (26.3%)	p=0.512^X^	20/26 (76.9%)	p=0.096^X^

t Independent (Student) t-test (between UR/PG project and published papers);

X Chi-squared;

±Standard deviation

## Discussion

The primary objective of this study was to compare the weighting of word counts for each IMRaD section between postgraduate (PG), undergraduate (UG), and published projects in Rwanda. We found that PG student dissertations used significantly more words than the published papers. This could be contributing, with other factors, to the low publication output of the PG students' research activities. We found that only four (10%) PG pediatric research projects were published between 2012-2017. In Germany one study found that of 807 research projects approved by the IRB, 52% were published [21]. The authors of this study found that study design was not significantly associated with subsequent publication but that multi-center status, international collaboration, and a large sample size were positively associated with subsequent publication [21]. In our study, 73% of PG research projects were cross-sectional (Table 1). However, in the German study [21], it was found that study methodology did not affect the proportion which were published, therefore in our population the high number of cross-sectional studies should not be affecting the publication rate. In Holland (2010) it was found that 15% of 2973 undergraduate students had published a piece of literature during their studies [22]. In our study, we were unable to identify how many UG projects were published. There are numerous challenges to publishing research findings in setting such as Rwanda, namely; lack of skills to write-up, supervision, time, motivation/desire, choosing and identifying the right peer-reviewed journal, navigation of the submission process, English language, and “publishing language” [9]. This can then be followed by the disheartening rejection of a manuscript during the peer-review process. In addition, some journals require either submission, type editing and/or publication fees, which most students cannot afford in the resource-limited setting. These challenges are surmountable and findings from Rwanda have shown that confidence and skills in academic writing can improve through training and intervention [14]. Researchers in Rwanda should remain committed to disseminating the results of all researches they conduct. Many students and residents may lack the skills required to overcome the barriers in navigating the peer-review process and therefore supervisors should provide significant support in achieving this goal. Regarding the writing style, literature has already demonstrated that undertaking research helps students develop critical thinking and fosters development of skills in scholarly activities [7, 10, 23]. The PG students used significantly more words (mean 5163) in order to write-up their research compared to the peer-reviewed articles (control group) (mean 2959, p<0.001). This could reflect a number of factors, namely the academic regulation dictated to them and the writing style of their predecessors.

All PG sections of the IMRAD were significantly longer in PG dissertations compared to peer-reviewed articles (control group). We were unable to find any published articles from other institutions regarding this subject. We are postulating that one possible explanation for low rates of research dissemination in our population is that dissertations are not concise and therefore the final write-up is not an appropriate length for submission to a peer-reviewed journal. Dissertations involve a major educational element to the student's experience. The dissertation is submitted solely by the student as part of their assessment with supervisors giving “guidance” rather than tight editing. For publication, however, multiple authors in clear collaboration, according to a clearly defined journal format, edit the text. These skills of writing concisely also need to be learnt by students and they also need guidance in order to be able to do so. Being able to write in a concise, academic, publishable style, is an additional skill and should not weaken, but rather strengthen, the dissertation required for graduation. The primary focus of students is to undertake a piece of work as a requirement of their studies. Publication is a secondary opportunity that some students will exploit and it is not a requirement for graduation. Research authors publish for personal and altruistic reasons that are both valid. Personal reasons include increasing their personal academic profile, for personal development, to better understand a topic, to build their reputation and for career advancement. Altruistic reasons include raising awareness of the topic and research, to disseminate findings, and ultimately improve quality of care for patients. Regarding the ethics of non-publication; research is dependent on the willingness of participants to expose themselves to the risks involved [24]. The ethical justification for these risks is that society will eventually benefit from the knowledge gained from the study. Researchers therefore have an ethical responsibility to report the results of research involving human subjects [25]. The Declaration of Helsinki (2014) states that; “researchers, authors, sponsors, editors, and publishers all have ethical obligations concerning the publication and dissemination of the results of research” [26]. Is there therefore an ethical challenge that students may undertake research for their own personal benefit (i.e. learning skills, graduation etc) without following-up to publish the results for the benefit of society and the scientific community? Both are important and need to be considered when implementing research activities into curricula. Likewise, institutions may have an ethical duty to ensure that students are given the skills to publish, in order to maximise the benefits to the scientific community. Medical students and residents have busy clinical duties, and when research is undertaken as a condition of graduation, it often occurs towards the end of studies. This makes returning to a dissertation at a later date to prepare it for submission less likely. Therefore, writing-up the dissertation in a publishable “manuscript” format, concisely, ready for journal submission could not only teach the student new writing skills but also increase the likelihood of the results being disseminated.

## Conclusion

This is the first study to assess the writing style of Rwandan medical students and pediatric residents. The results should be used to strengthen research activities in Rwanda and further afield in the Sub-Saharan region so that dissemination of research findings will be increased.

**Limitations:** We accessed 10% and 54% of UG and PG dissertations respectively and therefore our results may not be wholly representative of the whole cohort. We opportunistically selected the control group and this could have been subject to bias. A potential source of bias and confounding was the change in official language in Rwanda in 2008 from French to English. This may have affected conciseness of writing style of graduates who were primarily educated in French but needed to write-up their dissertations in English. Many of the UG projects could be described as “service review” providing useful information to local departments. Therefore, these may not be suitable for publication, however, students gaining the skills to write-up in publication style would still benefit the student for a future life as clinician-researchers.

**Implementation**: Writing concisely is a new skill for the students to learn and develops them for future academic and professional scientific writing. These results are particular to the academic regulations that students are given. Therefore, they need to be interpreted within the academic institution where this article is being read. If institutions, such as UR are committed to achieving their goals of increasing academic outputs then their doctors and specialists of the future need to be taught the necessary skills for concise academic writing. The findings of this study will not lead to an increase in the quality of published work. Rather it highlights the need to guide students to write within the expectations of peer-reviewed journals in order to increase the dissemination of the students' findings and increase the impact of the institution. A simple step would be to modify academic regulations so that student's write-up in “manuscript” form rather than “dissertation” format. If researchers can write-up a multi-center randomized-controlled trial within 3000 words then a student can and should be taught and supported to do the same for a dissertation. This might limit student to write-up more of their reflective experiences and supplementary data. However, there is no reason why this can't be presented within appendixes of a dissertation. Further changes would be to incorporate teaching on scientific writing skills for all students, develop faculty in order to improve the research capacity of the individual departments, and finally encourage ethics boards to ensure that research is disseminated.

### What is known about this topic

- Encouraging students to publish dissertation work is important as the future interpretation and synthesis of research, such as in systematic reviews, is compromised if journal publications represent a biased selection of the research work conducted;
- Many research projects go unreported and unpublished;
- Researchers have an ethical responsibility to report the results of research involving human subjects.

### What this study adds

- Postgraduate student dissertations used significantly more words than published papers and only 10% of postgraduate projects were published;
- Institutions who are committed to achieving their goals of increasing academic outputs must note that doctors and specialists of the future need training and guidance on concise academic writing;
- Some institutions may choose to modify academic regulations so that student's write-up in “manuscript” style and length rather than “dissertation” format.

## Competing interests

The authors declare no competing interests.
